# The Potential Impact of Childhood Traumatic Experiences on Coping Styles and Emotion Regulation of Nurse Practitioners During the COVID-19 Outbreak

**DOI:** 10.3389/fpsyg.2021.718780

**Published:** 2021-10-26

**Authors:** Liyuanke Wang, Yan Zhang, Xiaoli Zhang, Xiwang Fan, Luo Qiong, Chengping Hu

**Affiliations:** ^1^Suining Central Hospital, An Affiliated Hospital of Chongqing Medical University, Suining, China; ^2^Department of Psychiatry, Shanghai Pudong New Area Mental Health Center, Tongji University School of Medicine, Shanghai, China

**Keywords:** childhood traumatic experiences, coping styles, nurse practitioners, correlation, COVID-19

## Abstract

**Background:** During an epidemic of a novel infectious disease, frontline medical staff suffer from high psychological stress. Previous studies have found that traumatic childhood experiences are associated with mental and physical health in adulthood. Anxiety and depression were measured and analyzed in relation to childhood trauma and coping styles. This study aims to explore the correlational study between traumatic childhood experiences and coping styles among nurse practitioners.

**Method:** This study sampled 278 nurse practitioners from hospitals designated for the treatment of the novel coronavirus in Sichuan Province. The study measures included the Simplified Coping Style Questionnaire and the Childhood Trauma Questionnaire-Short Form. This research intends to use correlational study methods to explore the relationship between the two factors.

**Results:** Statistical analysis showed that there was no statistically significant difference in the general demographic data between the two groups.

**Conclusion:** Childhood traumatic experiences have a significant impact on the active coping of nurse practitioners, and active coping may be emotionally protective for nurse practitioners.

## Introduction

COVID-19 first occurred in late 2019 and has rapidly spread to most countries and regions around the world (Bchetnia et al., 2020). The sudden increase in diagnosed cases puts tremendous pressure on frontline medical staff, and nurse practitioners, as important clinical workers, work in a high-stress and high-intensity environment, which poses threats to their psychological well-being (Falcone et al., 2020; Xu et al., 2020). Front-line clinical staff mental health can have an impact on the physician-patient relationship (Marchini et al., 2021). Previous studies have found that traumatic childhood experiences are associated with mental and physical health in adulthood (Felitti et al., 1998; Anda et al., 2009). Additionally, coping styles have an impact on mental health (Ma et al., 2018). Therefore, this study aims to explore the correlation between traumatic childhood experiences and coping styles among nurse practitioners.

## Methods

This study sampled 278 nurse practitioners from hospitals designated for the treatment of the novel coronavirus in Sichuan Province. The study measures included the Simplified Coping Style Questionnaire (SCSQ), Self-Rating Depression Scale (SDS), Self-Rating Anxiety Scale (SAS), and Childhood Trauma Questionnaire-Short Form (CTQ-SF). CTQ-SF and a tool developed by the American clinical psychologist Bernstein to measure childhood abuse (Bernstein et al., 1997). There are 25 clinical entries and 3 validity entries for a total of 28 entries (Bernstein et al., 2003). The revised questionnaire introduced by Chinese researchers had five dimensions: emotional abuse, physical abuse, sexual abuse, physical neglect, and emotional neglect (Li et al., 2014). It has been shown that the Chinese version of the childhood trauma questionnaire had good reliability and validity in China (Xie et al., 2018). The study was approved by the ethical review committee of Suining Central Hospital. All nurse practitioners specify informed consent procedures. Statistical analysis was performed using the SPSS 21.0 software package (SPSS, Chicago, IL, United States).

## Results

Descriptive and statistical comparisons of the general characteristics of the study participants are shown in Table 1.

**TABLE 1:** General characteristics of the study participants.

Characteristics	All	Non-trauma group	Trauma group	*p*-value
Gender (male/female)	10/268	7/154	3/114	0.432
Age (years)	40.69	19.56 ± 2.012	19.62 ± 2.188	0.824
Grade				0.074
Technical secondary school	31	24	7	
Junior college	242	133	109	
University education	5	4	1	

*Data are presented as the mean ± SD.*

Statistical analysis showed that there was no statistically significant difference in the general demographic data between the two groups.

Significant differences in SAS, SDS and active coping scores were observed between the two groups (*p* < 0.001) in Table 2. However, the difference in passive coping style scores between the two groups was not statistically significant Table 2.

**TABLE 2:** Comparative analysis of coping styles and emotional scores in childhood trauma and non-trauma groups.

	Trauma group	Non-trauma group	*t*	*p*
SAS	41.92 ± 10.97	37.09 ± 6.02	−4.69	0.000
SDS	55.98 ± 6.19	52.56 ± 4.67	−5.24	0.000
Active coping	21.15 ± 7.30	25.02 ± 6.12	4.80	0.000
Passive coping	8.60 ± 4.26	8.83 ± 3.76	0.48	0.636

*SDS, Self-Rating Depression Scale; SAS, Self-Rating Anxiety Scale.*

There was a significant negative correlation between positive coping style and anxiety and depression in the trauma group, with correlation coefficients ranging from 0.3 to 0.5 Table 3. It is assumed that a positive coping style has a protective effect against anxiety and depression.

**TABLE 3:** Correlation of coping styles among the trauma group with anxiety and depression.

	Active coping	Passive coping
SAS	−0.36**	0.18*
SDS	−0.52**	0.09

**Correlation is significant at a confidence level of 0.01. *Correlation is significant at a confidence level of 0.05.

## Discussion

Childhood trauma experiences are negatively correlated with individual coping styles, and individuals with childhood trauma are less likely to develop positive psychological cues and positive coping styles (Edinger et al., 2020; Wan et al., 2020). Childhood trauma experiences can be biologically embedded in the brain, leading to long-term activation of the stress response system as well as anxiety and depression (Bellis et al., 2017; Bryan and Beitz, 2019). Coping styles refer to the way an individual perceives and behaves in response to an environment beyond his or her control due to internal and external factors (Flewelling et al., 2020; Rohleder et al., 2020). Passive coping styles, such as avoidance and fantasy, are associated with psychological symptoms such as anxiety and depression; individuals who adopt positive coping styles have stable mechanisms for regulating their emotions and are more likely to feel positive emotions (Jones et al., 2016; Gao et al., 2021). At the same time, previous studies have found that the protective effect of coping style on effects of war trauma exposure (Fino et al., 2020). The importance of emotion regulation skills especially in healthcare professionals like nurses who are routinely exposed to high stress and emotionally salient situations (Fino et al., 2021). The relevance of childhood traumatic experiences in terms of implications for psychosocial support programs in healthcare personnel (Fino et al., 2019).

## Conclusion

In summary, childhood traumatic experiences have a significant impact on the active coping of nurse practitioners, and active coping may be emotionally protective for nurse practitioners. The limits of the present study and state the need to verify these results in a more robust design of research.

## Data Availability Statement

The original contributions presented in the study are included in the article/supplementary material, further inquiries can be directed to the corresponding author.

## Author Contributions

CH and LQ contributed to the conception and design of the present study. LW recruited the patients and conducted the study. YZ, XZ, and XF undertook the statistical analyses and wrote the first manuscript draft. All authors contributed to the data analysis and drafting and revision of the article, gave final approval of the version to be published, and agreed to be accountable for all aspects of the work.

## Conflict of Interest

The authors declare that the research was conducted in the absence of any commercial or financial relationships that could be construed as a potential conflict of interest.

## Publisher’s Note

All claims expressed in this article are solely those of the authors and do not necessarily represent those of their affiliated organizations, or those of the publisher, the editors and the reviewers. Any product that may be evaluated in this article, or claim that may be made by its manufacturer, is not guaranteed or endorsed by the publisher.
